# Designing Mentorship for Constrained Systems: Reframing Workforce Development in Rural and Remote Health

**DOI:** 10.3390/ijerph23050676

**Published:** 2026-05-20

**Authors:** Shanshan Lin, Leah Pascoe, Grace Ward, Lynn Sinclair, Marlene Payk, Amy Zheng, David Sibbritt, Wenbo Peng

**Affiliations:** 1 Faculty of Health, University of Technology Sydney, Sydney, NSW 2007, Australia; lynn.sinclair@uts.edu.au (L.S.); amy.zheng@uts.edu.au (A.Z.);; 2 Rural Doctors Network, Newcastle, NSW 2065, Australia; 3 Diabetes Australia, Canberra, ACT 2612, Australia

**Keywords:** rural health workforce, mentorship, workforce development, cultural safety, health systems

## Abstract

**Highlights:**

**Public health relevance—How does this work relate to a public health issue?**

- Rural and remote health systems face persistent workforce constraints that affect the delivery of chronic disease care, including diabetes management.
- Mentorship is widely used to support health professionals, but existing models often do not align with the realities of constrained service environments.

**Public health significance—Why is this work of significance to public health?**

- This commentary reframes mentorship as a system-embedded approach that operates within routine care rather than as a separate training activity.
- It highlights how mentorship design must reflect service constraints, workforce capacity, and cultural context to remain feasible and effective.

**Public health implications—What are the key implications or messages for practitioners, policy makers and/or researchers in public health?**

- Integrating mentorship into everyday practice can support continuous workforce development without increasing service burden.
- System-aligned, co-designed mentorship models may strengthen workforce capability, particularly in Indigenous and resource-constrained settings.

**Abstract:**

Rural and remote health systems continue to face persistent workforce challenges that affect the delivery of chronic disease care, including diabetes management. Mentorship is widely recognised as a valuable strategy for supporting health professionals, with demonstrated benefits for practice development and workforce sustainability. However, many mentorship approaches are developed in well-resourced settings and assume stable infrastructure, protected time, and workforce capacity. These assumptions may not align with the realities of rural and remote practice, where service pressures and resource constraints shape everyday care. This commentary examines how mentorship can be designed for constrained health systems. It proposes a systems-oriented perspective that positions mentorship as part of routine practice rather than as a separate professional development activity. Emphasis is placed on flexibility, co-design, and cultural safety, with attention to how mentorship can be integrated within workforce development pathways. This reframing has implications for strengthening rural health services by supporting continuous, context-responsive learning within routine practice. More broadly, this approach offers a scalable pathway to workforce strengthening in geographically dispersed and resource-variable health systems.

## 1. Introduction

Health services in rural and remote settings operate under sustained workforce and service delivery pressure [1]. Workforce shortages, geographic distance, and service demand intersect in ways that shape both care delivery and professional support [2,3]. These conditions are particularly visible in the management of chronic diseases such as diabetes, where continuity of care and clinical confidence are critical. Health professionals in these environments often work with limited access to specialist input and fewer opportunities for ongoing development. Professional isolation is common, and support is not always available when it is most needed [4,5]. Under these conditions, workforce capability cannot be sustained through formal education or episodic training alone [2,6]. It must also be supported within the flow of everyday practice.

Mentorship is frequently proposed as a response to these challenges [7]. However, current mentorship models do not always align with the realities of rural practice [8,9]. This commentary argues that mentorship needs to be reconsidered, not as an additional activity, but as a form of system-embedded support that operates within the constraints of real-world clinical service environments. This Commentary does not report findings from a specific mentorship intervention, implementation study, or structured review. Instead, it offers a conceptual perspective on how mentorship can be designed to function within rural and remote health systems.

## 2. Mentorship in Rural Health: Contributions and Limitations

There is well-established evidence that mentorship supports professional growth. Across health professions, mentorship has been shown to strengthen clinical judgement, support transitions into new roles, and provide access to experiential knowledge [4]. In rural and remote contexts, where professional isolation and limited access to specialist support are common, these contributions are particularly valuable. Despite these benefits, many established mentorship models originate from well-resourced health systems. They often rely on assumptions such as stable schedules, reliable communication infrastructure, and the availability of experienced mentors who can commit protected time [2,3]. When applied to rural and remote settings, these assumptions are frequently difficult to sustain.

Workforce shortages, high service demand, and competing clinical priorities mean that participation in additional activities is often negotiated against immediate care needs for health professionals in rural and remote settings. Although adaptations have extended the reach of mentorship into rural contexts, including the use of digital platforms and more flexible delivery formats [10], many continue to focus on modifying modes of delivery. In this context, questions about how mentorship is expected to function under these conditions remain under-examined.

These conditions directly shape how mentorship needs to be designed. In settings where time is limited and service delivery takes priority, mentorship cannot depend on regular scheduling or sustained engagement. Instead, it must accommodate interruption, variability, and competing demands as part of everyday practice [2,3]. Models that continue to assume continuity and availability risk becoming misaligned with how care is delivered and difficult to sustain over time. Beyond these operational constraints, care in rural and remote settings is delivered across diverse communities, including Indigenous Peoples, where relationships, trust, and cultural context shape clinical practice [11,12]. Therefore, mentorship approaches that do not explicitly engage with cultural context may remain disconnected from the realities of care and limit their capacity to support culturally safe practice.

Seen in this light, the central issue is not the value of mentorship itself, but how it is configured. When design assumptions remain anchored in well-resourced environments, mentorship can struggle to function in settings where constraint, variability, and cultural context are defining features of everyday care. Addressing these conditions is thus important to ensuring that mentorship is not only conceptually sound but also practical within rural and remote health systems.

## 3. Reframing Mentorship as a System-Embedded Capability Strategy

This requires rethinking how mentorship is conceptualised. Rather than positioning it as an activity added to clinical work, mentorship can be embedded within everyday practice. This changes both how mentorship is understood and how it is designed. Within this approach, mentorship is not scheduled around practice but intertwined with it. Learning is supported through interaction, reflection, and shared problem-solving as part of routine care. A brief discussion following a complex case, a conversation between colleagues, or a moment of guided reflection could all function as mentoring.

As such, flexibility becomes essential. Engagement cannot rely on fixed formats or consistent availability, but must accommodate variation in workload, connectivity, and local conditions. This may involve brief case discussions, informal peer conversations, or asynchronous communication that allows health professionals in rural and remote settings to connect when workload and service demands permit. Designing mentorship in this way requires input from those working within the system [12], making co-design central to this approach. Co-design ensures that mentorship reflects local realities rather than external assumptions, allowing different forms of engagement to emerge, including peer interaction and shared learning approaches that extend beyond traditional one-to-one models. This is particularly important in contexts where care is shaped by community relationships and local knowledge systems. In many rural settings, including those serving Indigenous communities, effective practice depends on trust, continuity, and cultural context [12,13]. Mentorship that is responsive to these conditions can support not only clinical capability but also culturally safe practice.

## 4. From Individual Participation to Workforce-Level Capability

When mentorship is embedded in this way, it shifts from an optional activity taken up by individuals to a mechanism through which workforce capability is developed over time. This shift is particularly important in rural and remote settings, where experienced health professionals are already under pressure and reliance on a small number of mentors is neither equitable nor sustainable [5]. More distributed approaches enable knowledge to circulate across the workforce, reducing individual burden while supporting broader participation. Given the circumstances, mentorship is not only about immediate support but also about progression, supporting health professionals as they enter rural practice, build capability, and move into leadership roles. This positions mentorship as part of maintaining continuity within the workforce. Its contribution could be further strengthened when aligned with professional development structures. Linking mentorship to recognised competencies and organisational priorities allows it to become part of how the system operates, rather than an additional activity [3].

## 5. Implications for Practice, Policy, and Research

Extending this system-embedded view of mentorship, there are clear implications for how support is designed, governed, and sustained within rural health systems. In practice, this involves prioritising forms of support that are integrated within the delivery of care, allowing learning to occur without requiring disengagement from service responsibilities. Over time, such approaches may contribute to more consistent clinical decision-making and greater confidence in managing complexity, particularly in settings where access to specialist input is limited.

Embedding mentorship within routine workflows requires attention to how services are structured and supported. This includes recognising informal and practice-based mentoring between health professionals at different levels of experience as legitimate components of workforce development. Creating space for brief case discussion, reflective exchange, and peer consultation within existing meetings may provide a feasible pathway for integration without increasing workload burden. At a broader level, mentorship can be understood as part of workforce infrastructure rather than as a discrete or time-limited program [6,14]. Recognising mentorship in this way creates opportunities to align it with workforce strategies, accreditation requirements, and continuing professional development frameworks. Such alignment may support sustained engagement, reduce reliance on short-term initiatives, and distribute mentoring responsibilities more evenly across the workforce.

These considerations are particularly relevant for Indigenous health workforce development. Approaches grounded in co-design and cultural context can support not only clinical capability but also leadership development and culturally safe practice. When mentorship is shaped in partnership with Indigenous communities and health services, it has the potential to strengthen local workforce pathways, including transitions into specialised areas such as diabetes care, and to support models of care that reflect community priorities and knowledge systems.

This perspective also has relevance for workforce development initiatives in regional and Pacific contexts, where health systems often operate under similar constraints related to workforce capacity, geographic dispersion, and infrastructure variability. In these settings, mentorship that is integrated within routine practice and aligned with local service conditions may offer a feasible approach to supporting ongoing capability development without requiring substantial additional resources. Such approaches may be particularly valuable in strengthening education-to-practice pathways and supporting locally led models of care.

Future research should examine how these approaches influence practice over time, including their contribution to workforce stability, the development of clinical capability, and the delivery of culturally safe care. There is also value in exploring how such models can be adapted across different professional groups, health conditions, and system contexts where similar constraints shape workforce development.

## 6. Conclusions

Mentorship remains an important element of workforce development in rural and remote health. Its value is well recognised, but its design has not always kept pace with the conditions in which it is applied. Reframing mentorship as a system-embedded and practice-integrated approach offers a way forward. By aligning mentorship with the realities of constrained health systems, it can support ongoing learning without adding to existing service pressures. It also provides a pathway for strengthening workforce capability in settings where resources are limited and access to specialist support is constrained. Under this model, mentorship shifts from participation in a discrete program to a mechanism through which learning is sustained within everyday practice. This positions mentorship as part of how workforce capability is developed and maintained over time in real-world conditions.

## Data Availability

No new data were created or analysed in this study. Data sharing is not applicable to this article.
